# Detection and Genomic Characterization of Senecavirus A, Ohio, USA, 2015

**DOI:** 10.3201/eid2207.151897

**Published:** 2016-07

**Authors:** Leyi Wang, Melanie Prarat, Jeff Hayes, Yan Zhang

**Affiliations:** Ohio Department of Agriculture, Reynoldsburg, Ohio, USA

**Keywords:** Senecavirus A, SVA, idiopathic vesicular disease, detection, genomic characterization, viruses, Ohio, United States

**To the Editor:** Senecavirus A (SVA), formerly Seneca Valley virus, is a single-stranded positive-sense, nonenveloped RNA virus (*1*). The RNA genome of SVA is 7.2 kb long and is translated into a polyprotein in a host cell. The polyprotein is then posttranslationally cleaved into mature proteins, including 4 structural viral capsid proteins (VP 1–4) in the N terminus and 7 nonstructural proteins (2A, 2B, 2C, 3A, 3B, 3C^pro^, and 3D^pol^) in the C terminus (*1*). SVA was discovered as a contaminant of PER.C6 cells and is closely related to viruses in the genus *Cardiovirus* (*1*). Genomic characterization has led to classification of SVA in a new genus, *Senecavirus*, family *Picornaviridae*. A retrospective study conducted in the United States showed that the samples collected during 1988–2001 were SVA positive, and genetic analysis revealed that the sequences of all 7 SVA isolates are considerably similar to the first US SVA strain (SVV-001), suggesting that SVA may have been circulating in the US pig population for a long time (*2*).

Idiopathic vesicular disease (IVD) is a vesicular disease of pigs, and etiology is unknown (*3*). The clinical signs of IVD are fever, lameness, and vesicular lesions on various body parts including the oral cavity, snout, and coronary bands (*3*). Despite not being a debilitating disease, IVD is noteworthy because it causes lesions clinically indistinguishable from those of other vesicular animal diseases, including foot-and-mouth disease (FMD), vesicular stomatitis, swine vesicular disease, and vesicular exanthema of swine. IVD has been reported in several countries, including the United States (*4*–*7*), and has been recognized in several US states, including Florida, Indiana, and Iowa (*4*,*8*,*9*). Several lines of evidence show that SVA may be associated with IVD outbreaks in Canada, the United States, and Brazil (*3*,*7*,*10*). We describe the detection and genomic characterization of SVA isolated from pigs with vesicular lesions in Ohio.

In October 2015, the Animal Disease Diagnostic Laboratory of the Ohio Department of Agriculture received vesicle tissue, a vesicle swab sample, and whole blood from a sow with vesicular disease for rule-out testing for FMD virus (FMDV). The sow was lame on both front feet and had ruptured vesicular lesions on the snout and coronary bands of both front feet (Technical Appendix Figure). FMDV-specific real-time reverse transcription PCR was applied to the nucleic acid samples extracted from the 3 samples by using a MagMAX Pathogen RNA/DNA kit (Life Technologies, Carlsbad, CA, USA). All samples were negative for FMDV. We then performed 2 conventional reverse transcription PCRs with primers targeting 2 regions of the SVA genome (VP3/VP1, 3D/3′ untranslated region) on the same set of samples; the vesicle tissue and swab samples were SVA positive. Subsequently, we determined the whole-genome sequence of SVA by using 7 pairs of SVA–specific primers (Technical Appendix Table 1).

We completed sequencing the whole genomes for the vesicle tissue (SVA-OH1) and vesicle swab sample (SVA-OH2). On the basis of BLAST (http:blast.ncbi.nlm.nih.gov/Blast.cgi) searches, the SVA-OH1 and -OH2 isolates had 99% nt identity to 3 new US strains (USA/IA40380/2015, USA/SD41901/2015, USA/IA46008/2015) and 98% nt identity to 3 Brazil strains (SVV/BRA/MG1/2015, SVV/BRA/MG2/2015, SVV/BRA/GO3/2015) from GenBank. The Ohio isolates also shared 96% and 94% nt identity with a Canada strain (11-55910-3) and the first US SVA strain (SVV-001), respectively. Further analysis showed that, in comparison with these 8 strains with complete genome sequences available in GenBank, the 2 Ohio SVA isolates had 22 unique nucleotide mutations in the genome: 1 in the VP4 gene, 5 in VP2, 2 in VP3, 1 in VP1, 4 in 2B, 3 in 2C, 3 in 3A, 1 in 3B, and 2 in 3D (Technical Appendix Table 2). Among the 22 unique mutations, there were 2 nonsynonymous mutations at position 2082 in the VP3 gene of both isolates and position 5037 in the 3A gene of SVA-OH1 and 1 unique synonymous mutation only in SVA-OH2.

Phylogenetic analysis of the complete genome further supports that the 2 Ohio SVA isolates are closely related to each other and clustered together with the 3 recently isolated US strains, were less closely related to the isolates of the Brazil cluster, and were more distantly related to the isolate from Canada and the original SVA strain reported from United States (Figure). Consistent with the previous findings (*1*), all SVA isolates from different countries clustered together under the genus* Senecavirus*, which is most closely related to the genus *Cardiovirus* of the family *Picornaviridae* (Figure).

Our findings that a pig with clinical signs of IVD was infected with SVA and our genetic analysis demonstrating that the 2 Ohio SVA isolates are closely related to the other SVA strains from different countries provide further support for SVA involvement in IVD in pigs. More support could be provided by future studies, including continued surveillance of SVA and confirmation of the Koch postulates.

**Figure:**
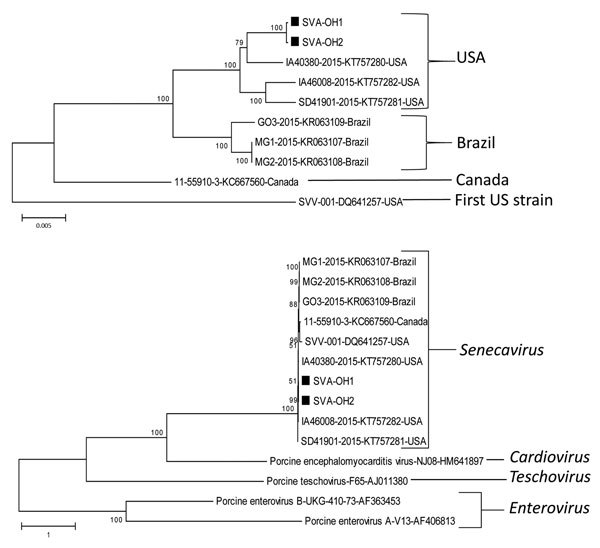
Phylogenetic trees constructed on the basis of the whole-genome sequences of isolates from the genera *Senecavirus* (SVA), *Cardiovirus*, *Teschovirus*, and *Enterovirus* of the family *Picornaviridae*, including the SVA-OH1 and -OH2 isolates (black squares) from pigs in Ohio, USA. Dendrograms were constructed by using the neighbor-joining method in MEGA version 6.05 (http://www.megasoftware.net). Bootstrap resampling (1,000 replications) was performed, and bootstrap values are indicated for each node. Reference sequences obtained from GenBank are indicated by strain name and accession number. Scale bars indicate nucleotide substitutions per site.

Technical Appendix Primers used for amplification and sequencing of the Senecavirus A complete genome, summary of unique mutations in SVA strains, and photographs of a sow with idiopathic vesicular disease.
