# Supplementary material for: Detection and Genomic Characterization of Senecavirus A, Ohio, USA, 2015
Source: Emerg Infect Dis. 2016 Jul;22(7):1321–3. doi: 10.3201/eid2207.151897 (PMC4918170; doi:10.3201/eid2207.151897)
Supplement: Technical Appendix — Primers used for amplification and sequencing of the Senecavirus A complete genome, summary of unique mutations in SVA strains, and photographs of a sow with idiopathic vesicular disease. [file 15-1897-Techapp-s1.pdf]

Article DOI: http://dx.doi.org/10.3201/eid2207.151897

Detection and Genomic Characterization of Senecavirus A, Ohio, USA, 2015

Technical Appendix

Technical Appendix Table 1. Primers used for amplification and sequencing of the SV-A complete genome*

Name	Sequence, 5′→3′†	Sense	Position‡	Amplicon size
P338	AACCGGCTGTGTTTGCTAGAGGC	Forward	59–81	
P339	gcgcTCTTCATTCAGCTCCTGTA	Reverse	1571–1589	1,531
P340	CAGGGAGGCCTCAACGGAGG	Forward	1393–1412	
P341	ACGGCGTCCTTCTCCAGTACCTT	Reverse	2824–2846	1,454
P342	TCTGCTGGTGAGGATTACAC	Forward	2623–2642	
P343	TTGGCATTGATCATAGTGGTGAGAC	Reverse	4184–4208	1,586
P344	GACCTAGTCAAAGAGGTGGTCGA	Forward	3958–3980	
P345	GCCCGGTCCGAGACGTACCATCA	Reverse	5447–5469	1,512
P346	CTTACACAGTCCGCTCTTCT	Forward	5281–5300	
P347	TCCACGTCATACGTGTTCTTTCT	Reverse	6511–6533	1,253
P348	gCTTAGACGGTGACTACTCTGA	Forward	6261–6281	
P349	TTTTTTTCCCTTTTCTGTTCCGACTGAGTTC	Reverse	7257–7287	1,027
P353	GGGGGCTGGGCCCTGATGCCCAG	Forward	10–32	
P355	GAGAACCATGTACTCATGGTGGTAGC	Reverse	466–491	482
P356§	TGGAAGCCATGCTCTCCTAC	Forward	7031–7050	Not applicable

*SV-A, Senecavirus A.
†Lowercase nucleotides are nonspecific sequence used to increase GC contents of the primers.
‡Positions correspond to SVV-001 strain (GenBank accession no. DQ641257).
§Primer used to sequence the 3′ end of SV-A.

Technical Appendix Table 2. Summary of unique mutations in SV-A strains*

Gene	No. mutations	Position†
VP4	1	902
VP2	5	1448, 1565, 1721, 1781, 1871
VP3	2	1988, 2082‡
VP1	1	3449
2B	4	3519§, 3524, 3746, 3788
2C	3	4163, 4442, 4688
3A	3	4961, 5037,‡¶ 5063
3B	1	4165
3D	2	6149, 6875
Total	22	Not applicable

*SV-A, Senecavirus A; VP, viral capsid protein.
†Positions correspond to strain SV-A-OH1 (GenBank accession no. KU058182).
‡Nonsynonymous mutation.
§Unique mutation only present in SV-A-OH2.
¶Unique mutation only present in SV-A-OH1.

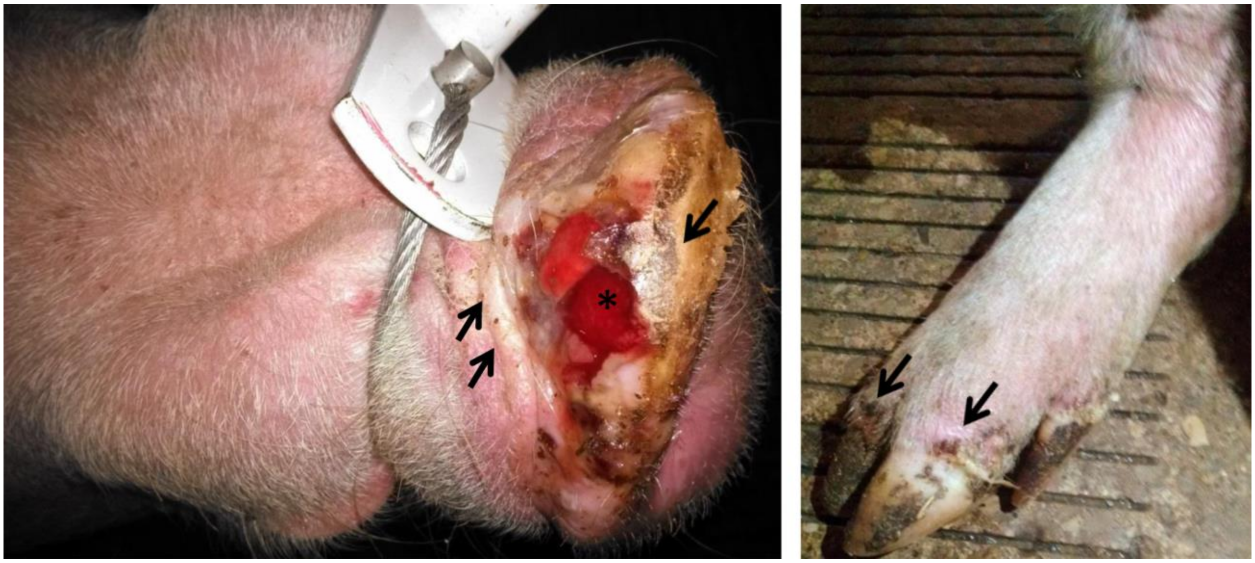

Technical Appendix Figure. Photographs of snout (left) and affected foot (right) of sow with idiopathic vesicular disease. Note ruptured vesicle on snout revealing red ulcerated region of exposed dermis (asterisk) with flaps of devitalized epidermis at margins (arrows). The affected foot on the right shows 2 focal ulcerations covered by brown scabs at the coronary band of each digit (arrows).
